# Curbing an outbreak of circulating vaccine derived poliovirus type 2 in Dollo Zone, Somali Region, Ethiopia: response to outbreak

**DOI:** 10.11604/pamj.2022.42.46.32856

**Published:** 2022-05-17

**Authors:** Legesse Kidanne, Filimona Bisrat, Mohammud Mohammed, Negussie Deyessa

**Affiliations:** 1 CORE Group Polio Project Secretariat, Addis Ababa, Ethiopia,; 2 Organization for Welfare and Development in Action, Jigjiga, Ethiopia,; 3 Department of Preventive Medicine, School of Public Health, College of Health Sciences, Addis Ababa University, Addis Ababa, Ethiopia

**Keywords:** Poliovirus, circulating vaccine, derived poliovirus, outbreak-response

## Abstract

**Introduction:**

although the oral polio vaccine prevents virus transmission from person to person, it is crucial for poliovirus eradication. The continued use of live attenuated poliovirus poses an ongoing risk of circulating Vaccine Derived Poliovirus-2 (cVDPV2) outbreaks. This study assesses the response to the cVDPV2 outbreak in Dollo Zone, Somali Region, Ethiopia.

**Methods:**

after examining and verifying the occurrence of the outbreak, a team was established and prepared by resource mobilization, advocacy, and social mobilization. The group endorsed a four-step vaccination strategy, first the rapid response within 14-days by vaccinating a monovalent oral poliovirus-2 (mOPV2) to all under 5-year children in the Zone. The team further enhanced Supplementary Immunization Activities (SIA) for all under-five children with repeated doses of vaccines. At the same time, the team initiated community-based surveillance of Acute Flaccid Paralysis (AFP).

**Results:**

in the rapid-response immunization, an average of 91.4% of 0-11 months old and 90.2% of 12-59 months children were vaccinated. In SIA-1, the team vaccinated an average of 88% and 97%, and in SIA-2, 94.8% and 97.6% of children 0-11 months old and 12-59 months old, respectively. The active community-based surveillance of AFP revealed the existence of the disease in a sporadic form, of which two cases were found in Bokh district.

**Conclusion:**

the response to curb the outbreak of cVDPV2 has shown a flow of actions to combat the outbreak. Strengthening and formation of response teams at different levels, resource mobilization, advocacy, and social mobilization are all essential components in maximizing the response to the outbreak.

## Introduction

Poliomyelitis was regarded as a severe disease with high disability and mortality in the early decades of modern medicine [1, 2]. In the past, polio had a high case fatality [3] and disability that affected the quality of life [4]. Furthermore, some early worries regarding vaccines causing death rate to be isolated post-vaccine introduction, although high-profile prior vaccine safety episodes [5]. Moreover, introducing different polio eradication strategies has eliminated wild poliovirus first in the developed continents and later in the most developed world [6-9].

Wild poliovirus types 2 and 3 were eliminated in September 2015 [10] and October 2019 [8], respectively. Type 1 wild poliovirus is still prevalent in two countries: Pakistan and Afghanistan [11, 12]. The administration of the oral polio vaccine (OPV) has protected millions of children from paralysis. OPV also prevents person-to-person transmission of the virus and is vital to eradicating polio [12]. However, continued use of live attenuated Sabin type 2 polioviruses in the trivalent Oral Polio Vaccine (tOPV) posed an ongoing risk of circulating vaccine-derived poliovirus 2 (cVDPV2) and vaccine-associated paralytic poliomyelitis [13]. In under-immunized communities, the live, weakened virus originally contained in OPV can genetically revert into a form that can cause paralysis if allowed to circulate, this is known as circulating vaccine-derived poliovirus (cVDPV) [14].

The cVDPV outbreaks are driven by several factors, including low-quality polio outbreak response; declining immunity in young children to the type 2 virus after countries switched from trivalent to bivalent oral polio vaccine (bOPV) for routine immunization in 2016; and insufficient regular immunization coverage [13]. Globally, cVDPV2 outbreaks account for more than 90% of cVDPV episodes [15]. In 2020, 1,037 cases of cVDPV2 were confirmed from 24 countries (data as of March 3^rd^, 2021), compared to 366 patients from 15 countries in 2019 [14]. The cVDPV2 outbreaks were initially discovered in October 2017 in Banadir Province, Somalia, and the causative virus was genetically related to the poliovirus causing Somalia, SOM-BAN-1 outbreak from July 2019 to February 2020. Polioviruses isolated from three AFP patients in Ethiopia and ten sewage samples from Banadir were genetically related to outbreaks. In Ethiopia, four new cVDPV2 emergences (ETH-ORO-1, ETH-ORO-2, ETH-ORO-3, and ETH-SOM-1) were confirmed from 15 AFP patients and through environmental surveillance in Addis Ababa and the Somali region [16, 17].

From May 20^th^ to November 3^rd^, 2020, a total of 37 VDPV2/ cVDPV2 cases and three positive environmental samples (cVDPV2) were reported in Ethiopia, with seven separate cVDPV2 outbreaks and three separate emergences [18]. Given the cross-border population movements across the Horn of Africa and subnational immunity and surveillance gaps, Somali, Oromia SNNPR, Dire Dawa, and Harari Regions are considered high-risk corridors for further outbreak transmission [19].

Due to the emergence of cVDPV2 in Bokh, outbreak response was carried out in the same way as for wild poliovirus outbreaks, with large-scale administration of OPV to rapidly boost population immunity and stop the cVDPV2. It is essential to study, analyse and document the three rounds of immunization campaign indicators, by defining the number by age group, the proportion of first-time vaccination local sound communications strategies, and stakeholders and interventions that worked to reach forgotten children successfully. Moreover, the study analyses Independent Monitoring (IM) data to assess the knowledge of mothers and vaccination status in selected clusters in the zone. Therefore, the purpose of this study is to assess the response to the circulating vaccine-derived poliovirus type 2 outbreaks in Dollo Zone, Somali Region, Ethiopia.

## Methods

**Study design:** the study conducted a descriptive design to describe the continuing vaccine-derived poliovirus (VDPV2) outbreak investigation and response in Dollo Zone, Somali Region, Ethiopia. The outbreak investigation is documented in Sufa *et al*. [18, 20], and the current study describes the actions taken to respond to the outbreak. Moreover, the study analysed secondary data collected in the rapid response, supplementary immunization activity within the Zone to determine the vaccination coverage, describing the coverage of monovalent oral polio vaccine type 2 (mOPV2) immunization campaigns conducted from May 25^th^ to June 17^th^, 2019.

**Study setting:** this study was conducted in Bokh woreda, Dollo Zone, Somali Region, Ethiopia. The Zone is one of the six Core Group Polio Project (CGPP) implementation Zones in the Somali Region. The boundaries of Dollo Zone include Qorahe and Jarar Zones in the West, Somaliland in the east, southeast, and north. The area is semi-arid, with limited rainfall throughout the year. The Zone has an estimated population of 450,413 predominantly pastoralist communities. There are 45,416 children under five years old, and 15,756 below one-year-old children. There are poor road infrastructures between the woredas and even worse between the Kebeles, inadequate access to safe water supply, and a lack of telecommunication networks. Most residents live in a typical “Somali traditional house” made of sticks, covered by old cloth tissues and plastic tents to protect them from the rain.

Four functional health centers and 18 functional health posts (only nine provide routine immunization) serve the Bokh Woreda population. Six health officers, four BSc nurses, thirteen clinical nurses, four midwives, three lab technicians, and 22 Health Extension Workers (HEWs) are the staff in the facilities. Woreda Health Office Head, one HEW (who is also the EPI and cold chain focal person), one health officer (who is the surveillance/public health emergency focal person), and a health management information systems (HMIS) specialist work at the woreda health office.

The woreda's surveillance system for acute flaccid paralysis is in place. The weekly reports were submitted by the woreda and health facilities equipped by a trained Public Health Emergency Management (PHEM) officer and a focal person at all health centers, supported by community-based surveillance partners such as Core Group Polio Project (CGPP) and Organization for Welfare and Development in Action (OWDA). The EPI coverage in the district based on health facility reports, and the Ethiopian Demographic Health Survey was 68.2% and 12% for Penta (OPV)-3, and 62.4% and 15% for measles, respectively [21].

**Response to the outbreak:** a detailed field investigation was done by using the standard WHO polio outbreak investigation checklist [22]. The index child was a 39-month-old female living in Angalo kebele of Bokh woreda, Dollo Zone, who had the onset of flaccid paralysis on 19^th^ May 2019. Three children in direct contact with the index case had stool samples positive for poliovirus type 2 (18), and WHO declared the outbreak on June 9^th^ 2019.

After verifying that the cases were caused by the polio vaccine-initiated virus (18, 20), a team comprising the WHO, UNICEF, OWDA, the Regional Health Bureau, and the local health and administrative offices were established and strengthened to further investigate and respond to the outbreak. The team prepared and acted a community mobilization, advocacy, and social mobilization related to the investigation and response in the district, the Zone, and the adjacent zones.

In collaboration with UNICEF, WHO, and OWDA, the Regional Health Bureau facilitated advocacy, social mobilization, and campaign launching at Bokh Woreda. The officials launched the campaign by administering the mOPV2 drops to children under five years old. The community mobilization was made using different media such as Jumma-prayer at the mosques, megaphones, and house-to-house mobilization by training and deploying community volunteers.

It also coordinated Zonal and Woreda Command Post Meetings, which discussed the daily performance of woredas, kebele, and vaccine retrieval status, the cold chain, transportation arrangements, and challenges faced. Problems encountered and raised during the meeting were discussed, and corrective actions were undertaken based on the issues identified. The zonal command post mapped the available vehicles and assigned them to the priority woredas to transport vaccinators, their supervisors, vaccines, and other supplies to the kebele and village levels. The team trained supervisors, and vaccination teams focused on vaccine management, the campaign, and daily team supervision and reporting. Further review was made on the tally sheets, report formats, and supervision checklist. The team also organized a campaign of monitoring and supervision to ensure the high quality of the vaccination campaign, and corrective measures were given accordingly.

**Figure 1:**
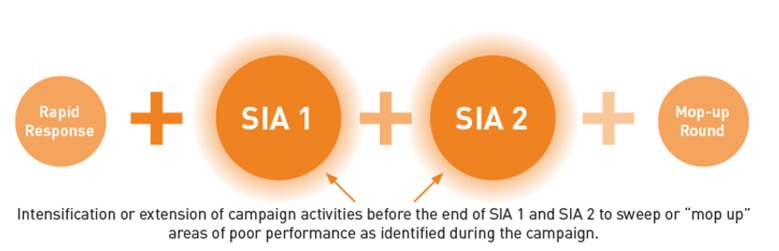
visual representation of timing and scale of immunization activities required, adopted from [22]

The response team endorsed a four-step vaccination strategy by GPEI for outbreaks and responding to poliovirus of any variant [23]. The team prompted the rapid response (RR), from June 22^nd^ to 25^th^ 2019, by vaccinating all under 5-year children in the Dollo Zone with a monovalent oral poliovirus-2 (mOPV2) where cases of cVDPV2 were found. Moreover, the team expanded the mOPV2supplementary immunization activities (SIA-1 and SIA2) to adjacent zones and continued vaccinating all under-five children, more than a month apart. However, mop-up was not recorded in this response (Figure 1).

Furthermore, through training of community volunteers, Core Group Polio Project through its partner OWDA has enhanced the weak Acute Flaccid Paralysis (AFP) surveillance by motivating community volunteers to conduct active community-based surveillance in the zone. The vaccination campaign was monitored and evaluated using independent monitors led by Jigjiga University in selected clusters *(woredas)* within Dollo Zone.

**Participants:** the source population for the data was all under-five children eligible for a vaccination against poliovirus through supplemental immunization campaigns, within health facilities or outreach sites, in Dollo Zone, Somali Region, Ethiopia. The study population was secondary data of under-five children retrieved from health facilities, health posts, and health centers in the health facilities of the zones. Aggregated data of children vaccinated for the rapid response and supplementary immunization activity one and two was retrieved.

**Variables:** the study included aggregated data for each round of vaccination. It included the history of past immunization, age in months, and place (district and Zone).

**Data sources and measurements:** the study retrieved secondary data registered in a Microsoft Excel datasheet from all health facilities in the Woreda and Zones that describe the immediate outputs of the epidemic. There were three rounds of vaccination, the rapid response, and the two supplementary immunization activities 1 and 2. Children´s history of immunization was registered as children were vaccinated for the first time in each round. The study also measured age in months and was categorized into (0 to 11) and 12-59 months, and by place (district and Zone). However, this study included a response to the outbreak in the Dollo Zone.

**Study size:** the inclusion of all children under five made the study not determine the sample size. The mobile nature of the population (pastoralists), and lack of knowledge of the exact date of birth of a child by some mothers have resulted to change in the known size of children. Here, the group has taken the largest size in any of the rounds as the target population.

**Statistical methods:** the secondary data from the rapid response and the two supplementary immunization activities collected in Excel format were assessed based on the eligible population, from zero to eleven months and 12 to 59 months old, for the Zone and district where the outbreak occurred. After exporting the data into SPSS for Windows, the investigators analysed the number and proportion of newly and formerly vaccinated children in the routine immunization of the three vaccination regimens. Furthermore, the data were assessed for children between 0-11 months and 12-59 months. For the independent monitoring data, the proportion of vaccination coverage and awareness of the caregivers´ campaign was assessed for each Woredas in the Zone. The study also assessed the findings of active community-based surveillance.

## Results

In response to the cVDPV2 outbreak, a team at the district, Zone, Region, the MOH, and the World Health Organization responded following the verification of the presence of a circulating poliovirus case. The team immediately initiated the rapid response by immunizing all children between the ages of 0 to 59 months, first in the Dollo zone, followed in the nearby neighbourhood zones. The House-to-House search was the preferred strategy to achieve the objective of reaching all target children. In addition, the team visited Quran schools, Kindergartens, Hospitals, Health Centers, water points, streets, markets, and border crossing points to maximize immunizing more children.

In the first rapid-response immunization, the team vaccinated 60,917 children (91.4% and 90.2% of children in the age group between 0-11 and 12-59 months, respectively). The coverage ranged from 70% (in Lehelyub) to about 97% (in Galadi) for children aged 0-11 months. The immunization coverage for children aged12-59 months ranged from 83% (in Wardher) to 94% (in Bokh and Galhamur) districts. (Table 1).

**Table 1:** rapid response of mOPV-2 vaccination status of 0 -11 and 12-59 months in Dollo Zone, Somalia region, Ethiopia

Woreda	Target (0-5 years)	Vaccinated	Percent of the target
**0-11 months**			
Bokh	3,709	3,469	93.5
Danod	2,329	2,079	89.3
Daratole	1,111	1,034	93.1
Galadi	4,574	4,422	96.7
Galhamur	1,806	1,688	93.5
Lehelyub	1,635	1,159	70.9
Warder	2,321	2,137	92.1
Zonal Total	17,484	15,988	91.4
**11-59 months**			
Bokh	14,030	13,248	94.4
Danod	9,452	7,902	83.6
Daratole	4,628	4,138	89.4
Galadi	18,138	1,6747	92.3
Galhamur	7,066	6,671	94.4
Lehelyub	4,648	4,247	91.4
Warder	9,555	7,964	83.3
Zonal Total	67,517	60,917	90.2

Similarly, the second round of immunization, the supplementary immunization one, was introduced a month later to immunize children under five in the Zone and neighbouring zones. In the first round of immunization, the supplementary immunization activity one (SIA-1), the team vaccinated an average of 88% and 97% of children in the age group between 0-11 and 12-59 months, respectively. The average immunization coverage for SIA-2 for 0-11 months of children was 94.8%, while for 12-59 months, it was 97.6%. The coverage for 0-11 months, ranged between 57% and 100% for SIA-1, and 87.5% to 100.0% for children 12-59 months. The immunization coverage for children aged 12-59 months ranged between 92-100% for SIA-1 and SIA-2 (Table 2).

**Table 2:** supplemental immunization activity one and two for mOPV-2 vaccination status of 0-11 and 12-59 months in Dollo Zone, Somalia region, Ethiopia

Woreda	Target	SIA-1	SIA-2
0-11 months		# (%)	# (%)
Bokh	3709	3,709 (100.0)	3296 (88.9)
Danod	2329	1,816 (78.0)	2329 (100.0)
Daratole	1111	640 (57.6)	1,001 (90.1)
Galadi	4574	4,272 (93.4)	4,490 (98.2)
Galhamur	1806	1,449 (80.2)	1,700 (94.1)
Lehelyub	1,635	1,635 (100.0)	1,431 (87.5)
Warder	2,321	1,869 (80.5)	2,321 (100.0)
Zonal Total	17,484	15,390 (88.0)	16,568 (94.8)
**11-59 months**		**# (%)**	**# (%)**
Bokh	14.030	12,909 (92.0)	14,030 (100.0)
Danod	9,452	8,867 (93.8)	9,452 (100.0)
Daratole	4,628	4,629 (100.0)	4,295 (92.8)
Galadi	18,138	18,138 (100.0)	17,205 (94.9)
Galhamur	7,066	7,066 (100.0)	6,710 (95.0)
Lehelyub	4,648	4,382 (94.3)	4,648 (100.0)
Warder	9,555	9,467 (99.1)	9,555 (100.0)
Zonal Total	67,517	65,457 (96.9)	65,895 (97.6)

**Figure 2:**
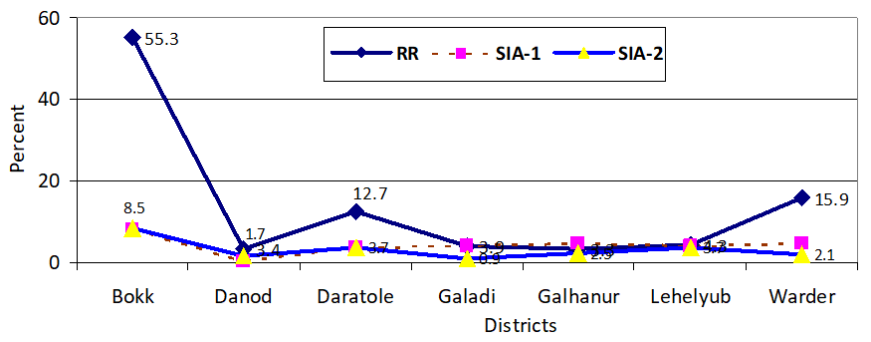
first-time vaccination during a rapid response, supplementary immunization one and two response of children between 0-11 months in Dollo Zone, Somalia region, Ethiopia

Although the outbreak response gave children a supplement to a previous vaccination, it also gave the mono-poliovirus immunization for the first time in three preventive rounds. During the rapid response, the study found that about 17% of children aged 0-11 months received vaccination for the first time. This first-time vaccination was as high as 55% in Bokh, about 16% in Warder, and 13% in Daratole. The average first-time coverage goes down to 4.7 in the SIA-1 and 3.3 in SIA-2 (Figure 2).

**Figure 3:**
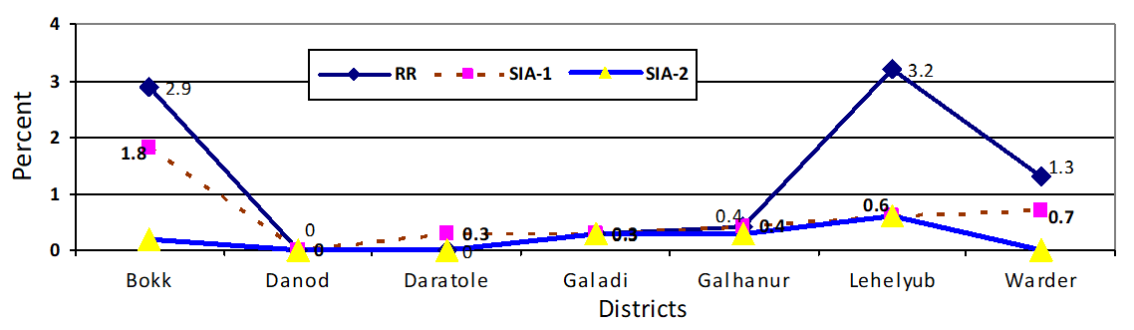
first-time vaccination during a rapid response, supplementary immunization one and two of children between 12-59 months in Dollo Zone, Somalia region, Ethiopia

Although few children aged 12-59 months received first-time vaccination, the study found an average of 1.6% in the first rapid response, 0.6% in the SIA-1, and 0.2% in the SIA-2. The magnitude of first-time vaccination during the rapid response round among 12-59 months was 3.2% in Lebeyub and 2.9% in Bokh districts. The coverage of first-time vaccination during the SIA-1 was still high among 12-59 months, especially in Bokh, with a 1.8%. However, coverage of first-time vaccination reduced to be below 1% in the SIA-2 (Figure 3).

**Figure 4:**
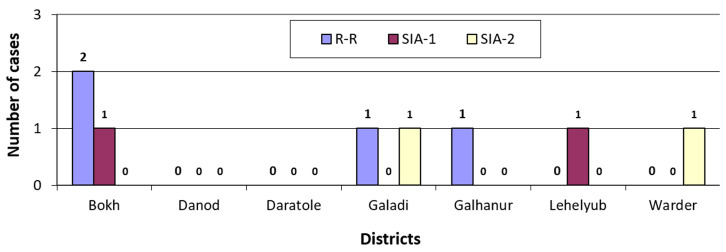
community-based surveillance of Acute Flaccid Paralysis in the age group 0-5 years during the rapid-response, SIA-1, and SIA-2, by districts in Dollo Zone, Somalia region, Ethiopia

The study illustrates the community surveillance of acute flaccid paralysis in the Zone during the three rounds of the outbreak response in Figure 4. During the rapid response immunization, the surveillance found four AFP cases: two cases in Bokh, one in Galadi, and another in Galhanur. Similarly, it found two cases in SIA-1, one in Bokh and another in Lehelyub, and the other two additional cases in SIA-2, in Galadi and in Warder (Figure 4).

The independent monitoring group from Jigjiga University found that 80% of children had a fingermark that showed they had been vaccinated against poliovirus type 2, and about 88% of their care providers of children were aware of the immunization campaign. The vaccination coverage rate was as low as 63% in Danot and as high as 92% in Bokh Woreda. Moreover, caregivers' awareness of the campaign's presence was adequate; which was 85% in Danoot and 92% in Bokh, where the outbreak occurred (Table 3).

**Table 3:** vaccination status and parents aware about the campaign among 60 randomly selected children each from three clusters (districts) selected by independent monitors in Dollo Zone, Somali Region, Ethiopia

Districts Observed 60 children (from each cluster)	Parents aware of the campaign Number (Percent)	Vaccinated (finger marker) Number (Percent)
Bokh	55 (91.7)	55 (91.7)
Danot	51 (85.0)	38 (63.3)
Galhamur	= =	51 (85.0)
**Total**	106 (88)*	**144 (80.0)**

* Denominator was of two districts (IM)

## Discussion

This study described the response to the outbreak of circulating vaccine-driven poliovirus Type 2 in Bokh district. The strengthening and formation of response teams at different levels of localities were important outputs to managing the response to the outbreak. This strengthening and formation of a response team at different levels of the administrative hierarchy were consistent with other outbreak responses, as the first reaction to an episode of an epidemic of a public health significance [24]. After establishing the response team, resource mobilization, advocacy, community, and social mobilization will enhance the coverage of the preventive action, in this case, immunization of children under-five years old. In this scenario, such resource mobilization, advocacy, and social mobilization may be the reason for the high vaccination coverage among children under five years in remote, challenging, and hard-to-reach areas, consistent with other studies [25, 26].

The immunization campaign started through rapid-response vaccination within 14 days of the laboratory-based verification and has covered a substantially high proportion of children. Experts and the World Health Organization recommended the starting time of the immunization campaign within 14 days of a laboratory declaration for a poliovirus outbreak [23]. The study found that the rapid response vaccinated about 55% of 0-11 months and 2.9% of 12-59 months for the first time in Bokh district, showing the community had a weak immunization program. This high number of never vaccinated children of 0-11 months old in the district during the outbreak shows that there were unreached children through routine immunization due to weak EPI services in the community. Similarly, the Penta 3 coverage rate was 65% in 2012, but the rate declined to 12% in 2017 in the district [18]. The EPI coverage in the community was 68.2% and 12% for Penta (OPV)-3, and 62.4% and 15% for measles based on health facility report and the Ethiopian Demographic Health Survey respectively [21]. The relatively high vaccination coverage data during the rapid and the two supplementary immunization activities can create high herd immunity that could break the circulating poliovirus [27].

## Conclusion

The response to curb the outbreak of circulating vaccine-driven poliovirus type 2 in Bokh district has shown the flow of actions to combat the episode. The strengthening and formation of response teams at different levels of administrative localities, resource mobilization, advocacy, and social mobilization are essential components that maximized the response to hinder the poliovirus epidemic. The researchers want to recommend the existence of community-level outbreak preparedness and rapid response team at all levels of the administration that should strengthen the expanded immunization program and the surveillance system in the community.

### What is known about this topic

- There is no well-documented outbreak response for circulating vaccine-derived poliovirus type-2 (cVDPV2) in hard to reach and pastoralist community;
- Difficult to create well-coordinated teamwork, communication, and stakeholders´ engagement in pastoralist setup;
- A large proportion of first-dose children during the rapid response is additional information to witness the immunity gap among the targeted children.

### What this study adds

- The study documented the practical application of the outbreak response theory recommended by World Health Organization (WHO);
- Implementation of three rounds of mOPV2 SIA can reach the unreached children in the pastoralist community and able to curb the circulating VDPV type-2 in the zone.

## References

[1] Groce NE, Banks LM, Stein MA (2014). Surviving polio in a post-polio world. Soc Sci Med.

[2] Miller ER, Moro PL, Cano M, Shimabukuro TT (2015). Deaths following vaccination: what does the evidence show?. Vaccine.

[3] Doshi SJ, Sandhu HS, Venczel LV, Hymbaugh KJ, Deshpande JM, Pallansch MA (2011). Poliomyelitis-related case-fatality ratio in India, 2002-2006. Clin Infect Dis.

[4] Ahlström G, Karlsson U (2000). Disability and quality of life in individuals with postpolio syndrome. Disabil Rehabil.

[5] Gee J, Weinbaum C, Sukumaran L, Markowitz LE (2016). Quadrivalent HPV vaccine safety review and safety monitoring plans for nine-valent HPV vaccine in the United States. Hum Vaccin Immunother.

[6] Nathanson N, Kew OM (2010). From emergence to eradication: the epidemiology of poliomyelitis deconstructed. Am J Epidemiol.

[7] Aylward B, Tangermann R (2011). The global polio eradication initiative: lessons learned and prospects for success. Vaccine.

[8] Leke RGF, King A, Pallansch MA, Tangermann RH, Mkanda P, Chunsuttiwat S (2020). Certifying the interruption of wild poliovirus transmission in the WHO African region on the turbulent journey to a polio-free world. Lancet Glob Health.

[9] Adebisi YA, Prisno III, DE-L, Nuga BB (2020). Last fight of wild polio in Africa: Nigeria´s battle. Public Health Pract (Oxf).

[10] Morales M, Tangermann RH, Wassilak SG (2016). Progress toward polio eradication—worldwide, 2015-2016. Morbidity and mortality weekly report.

[11] Angez M, Shaukat S, Alam MM, Sharif S, Khurshid A, Zaidi SSZ (2012). Genetic relationships and epidemiological links between wild type 1 poliovirus isolates in Pakistan and Afghanistan. Virol J.

[12] Kew OM, Wright PF, Agol VI, Delpeyroux F, Shimizu H, Nathanson N, Pallansch MA (2004). Circulating vaccine-derived polioviruses: current state of knowledge. Bull World Health Organ.

[13] Diop OM, Asghar H, Gavrilin E, Moeletsi NG, Benito GR, Paladin F (2017). Virologic monitoring of poliovirus type 2 after oral poliovirus vaccine type 2 withdrawal in April 2016 worldwide, 2016-2017. MMWR Morbidity and mortality weekly report.

[14] Initiative GPE: GPEI strategy for control of cVDPV2. 2020-2021.

[15] Wassilak S, Pate MA, Wannemuehler K, Jenks J, Burns C, Chenoweth P (2011). Outbreak of type 2 vaccine-derived poliovirus in Nigeria: emergence and widespread circulation in an underimmunized population. J Infect Dis.

[16] Alleman MM, Jorba J, Greene SA, Diop OM, Iber J, Tallis G (2020). Update on vaccine-derived poliovirus outbreaks worldwide, July 2019-February 2020. Morbidity and Mortality Weekly Report.

[17] Persons IVIA, County WIDC, Virginia W Update on Vaccine-Derived Poliovirus Outbreaks Worldwide July, 2019 February 2020. Notes 2020.

[18] Sufa D, Kerebih M, SheAbdi M, Belete H, Balalay V, al NBe (2019). Investigation Report of cVDPV2 Outbreak in Bokh Woreda of Dollo Zone. Somali Regional State.

[19] Dowdle WR, De Gourville E, Kew OM, Pallansch MA, Wood DJ (2003). Polio eradication: the OPV paradox. Rev Med Virol.

[20] Sufa D, Gerema U (2020). Investigation Report of cVDPV2 Outbreak in Bokh Woreda of Dollo Zone, Somali Regional State, Ethiopia. Case Rep Infect Dis.

[21] Sufa D, Kerebih M, SheAbdi M, Belete H, Balalay V (2019). al NBe: cVDPV Outbreak Investigation Report, Bokh Woreda, Dollo Zone, Somali Region, Ethiopia. In Edited by report D.

[22] (1991). World Health Organization: Responding to A suspected Polio Outbreak: Case Investigationm Surveillance and Control; A Manager's Checklist. In Edited by Immunization EPo.

[23] Organization WH (2020). Standard operating procedures: responding to a poliovirus event or outbreak.

[24] World Health Organization Geneva The global epidemiology of infectious diseases.

[25] Carvalho AF (2018). Advocacy for Stronger Immunization Programs. Vaccinology.

[26] Flores A, Villeda JA, Rodríguez-Fernández R, Chévez AE, Barrera L, Tezaguic R (2011). Advocacy and resource mobilization for rubella elimination in Guatemala. J Infect Dis.

[27] Cairns W, Smith S, Small R, Dunkerley R, Green D (1982). Herd immunity to poliovirus in Dundee. Health Bull (Edinb).

